# Digital analysis of external fixation area of proximal humerus fractures in elderly patients

**DOI:** 10.1186/s12891-021-04826-0

**Published:** 2021-11-26

**Authors:** Baorui Xing, Yadi Zhang, Xiuxiu Hou, Yunmei Li, Guoliang Li, Guangpu Han

**Affiliations:** Cangzhou Hospital of integrated traditional Chinese medicine and Western medicine, No.31, West Huanghe Road, Yunhe District, Cangzhou, 061000 Hebei Province China

**Keywords:** Elderly, Proximal humerus, 3D model, Anatomic measurement, Simulated screw placement

## Abstract

**Introduction:**

The purpose is based on anatomical basis, combined with three-dimensional measurement, to guide the clinical repositioning of proximal humeral fractures, select the appropriate pin entry point and angle, and simulate surgery.

**Methods:**

11 fresh cadaveric specimens were collected, the distance of the marked points around the shoulder joint was measured anatomically, and the vertical distance between the inferior border of the acromion and the superior border of the axillary nerve, the vertical distance between the apex of the humeral head and the superior border of the axillary nerve, the vertical distance between the inferior border of the acromion and the superior border of the anterior rotator humeral artery, and the vertical distance between the apex of the humeral head and the superior border of the anterior rotator humeral artery were marked on the 3D model based on the anatomical data to find the relative safety zone for pin placement.

**Results:**

Contralateral data can be used to guide the repositioning and fixation of that side of the proximal humerus fracture, and uniform data cannot be used between male and female patients. For lateral pining, the distance of the inferior border of the acromion from the axillary nerve (5.90 ± 0.43) cm, range (5.3-6.9) cm, was selected for pining along the medial axis of the humeral head, close to the medial cervical cortex, and the pining angle was measured in the coronal plane (42.84 ± 2.45)°, range (37.02° ~ 46.31°), and in the sagittal plane (28.24 ± 2.25)°, range (19.22° ~ 28.51°). The pin was advanced laterally in front of the same level of the lateral approach point to form a cross-fixed support with the lateral pin, and the pin angle was measured in the coronal plane (36.14 ± 1.75)°, range (30.32° ~ 39.61°), and in the sagittal plane (28.64 ± 1.37)°, range (22.82° ~ 32.11°). Two pins were taken at the greater humeral tuberosity for fixation, with the proximal pin at an angle (159.26 ± 1.98) to the coronal surface of the humeral stem, range (155.79° ~ 165.08°), and the sagittal angle (161.76 ± 2.15)°, with the pin end between the superior surface of the humeral talus and the inferior surface of the humeral talus. The distal needle of the greater humeral tuberosity was parallel to the proximal approach trajectory, and the needle end was on the inferior surface of the humeral talus.

**Conclusion:**

Based on the anatomical data, we can accurately identify the corresponding bony structures of the proximal humerus and mark the location of the pin on the 3D model for pin placement, which is simple and practical to meet the relevant individual parameters.

## Introduction

Proximal humerus fractures are the second most common type of fracture in the upper extremity, and about 10% of all fracture patients over 65 years of age have proximal humerus fractures [1, 2]. Treatment of older patients is often more difficult than in younger patients due to the presence of osteoporosis, osteoarthritis and rotator cuff injuries, and various systemic comorbidities [3, 4]. Conservative treatment of displaced or unstable fractures has led to poor functional recovery in a significant proportion of patients after surgery [5, 6]. At present, open reduction internal fixation is still one of the main surgical treatment methods [7, 8]. Shoulder arthroplasty is of course an option for older patients. Iacobellis suggests that reverse shoulder arthroplasty is an option for patients over 65 years of age with 3- or 4-part complex proximal humeral fractures. Although reverse shoulder arthroplasty is more invasive and more annular to the periarticular bone. However, it can provide good stability and a greater range of motion [9]. The blood supply to the proximal humerus mainly relies on the spinohumeral artery, and open reduction internal fixation and reverse shoulder arthroplasty tend to further damage the blood supply artery to the proximal humerus, increasing the risk of ischemic necrosis of the humeral head and damage to the axillary nerve [10–12]. External fixation can be used in poor bone and soft tissue conditions or as a rapid, minimally invasive method in patients with poor general condition or multiple injuries. Studies have shown that in young patients with good bone quality, bone strength is positively correlated with the effect of percutaneous pin fixation, but elderly patients also have a better prognosis after percutaneous pin fixation because they do not require high joint function and do not need to fully restore joint function, but can reduce shoulder pain and perform some simple daily activities [11, 13]. We believe that the application of external fixation for Neer II and III proximal humerus fractures in the elderly can also achieve good results.

The aim of this study is to indirectly measure the anatomical parameters of patients with proximal humeral fractures to select relatively safe nail entry points and angles. Simulate the surgery and develop an individualized external fixation surgical plan. It is an important reference for improving surgical outcomes and safety.

## Materials and methods

11 cadavers, 22 fresh shoulder specimens (All study shoulder joint specimens were provided by Cangzhou Hospital of integrated traditional Chinese medicine and Western medicine.), diagnosed with trauma and cardiovascular disease, aged 65-83 years, 4 female and 7 male. Inclusion criteria: (1) all specimens had no history of shoulder trauma or surgery; (2) all specimens had no history of primary or metastatic tumors of the proximal humerus and no history of chronic disease. All shoulder joint specimens for the study were provided by Cangzhou Hospital of integrated traditional Chinese medicine and Western medicine.

23 CT scan images of the shoulder joint in healthy elderly people. 14 males and 9 females, 46 shoulders. Developmental deformities of the shoulder joint and humerus, and bone tumors of the shoulder joint and humerus were excluded. The scan area was from the head of the superior border of the acromion to the distal humerus region. The image data were exported through the CT workstation and saved in DICOM format.

### Autopsy

**Fig. 1:**
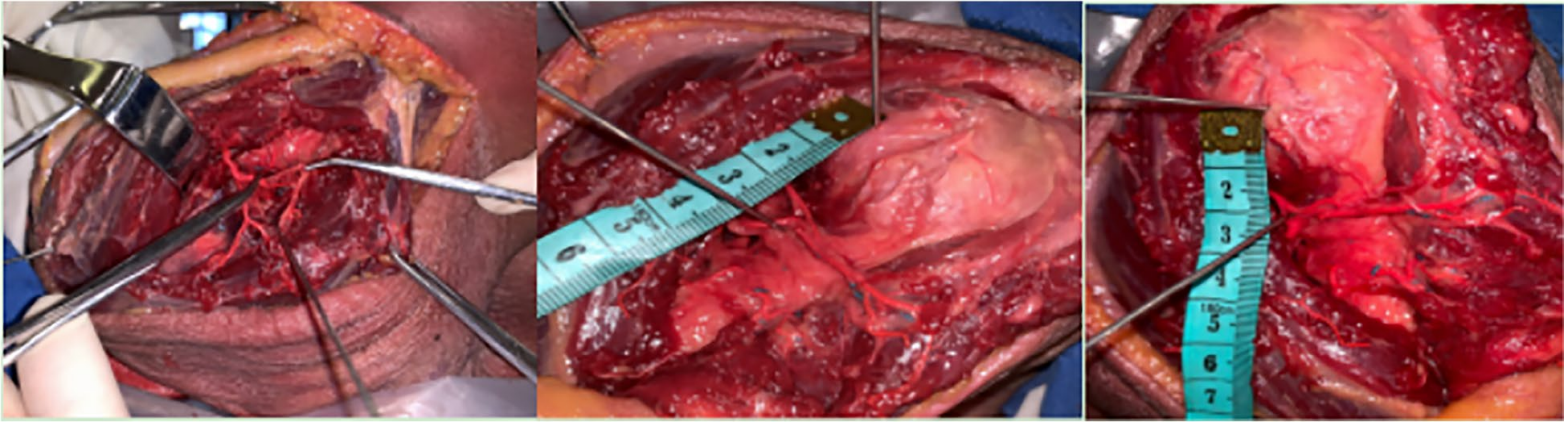
The route of the anterior humeral artery was exposed through an anatomical incision. The distance between the humeral head and the anterior humeral artery and the distance between the humeral head and the axillary nerve were measured

The cadaveric specimen was placed in neutral position at the shoulder joint. A longitudinal incision is made from the apex of the acromion downward, lateral to the shoulder joint, at the level of the acromion - deltoid stop. The skin is turned laterally to expose the subcutaneous tissue layer by layer. The deltoid muscle and fascia were exposed by separating along the direction of the deltoid fibers and from the crest of the shoulder downward. The deltoid fascia was incised along the anterolateral 1/3 of the deltoid muscle, and the anterolateral deltoid interosseous ridge was peeled off and exposed, along which the ridge was separated, and the medial side of the deltoid muscle was deeply explored with the fingers to determine the location and alignment of the axillary nerve. Subsequently, the middle deltoid muscle was split longitudinally, paying attention to the blunt detachment with gentle movements. During the process, the distribution and alignment of the neurovascular in the deltoid muscle were carefully observed, and the location of the axillary nerve trunk was combined to further understand the distribution pattern of the nerve in the proximal humerus. The deltoid incision was pulled apart on both sides with a orthopedic retractor, and the anterior and posterior spinohumeral arteries and axillary nerve were dissected and separated, and marked for protection. The upper end of the incision was extended, the deltoid acromion stop was dissected away and relocated to expose the important bony landmarks of the proximal humerus. Measure: 1) the vertical distance from the apex of the humeral head to the superior border of the axillary nerve; 2) the vertical distance between the anterior inferior border of the acromion and the superior border of the axillary nerve; 3) the vertical distance between the apex of the humeral head and the superior border of the anterior rotator humeral artery; 4) the vertical distance between the inferior border of the acromion and the superior border of the anterior rotator humeral artery (Fig. 1).

### Digital measurement of anatomical parameters

**Fig. 2:**
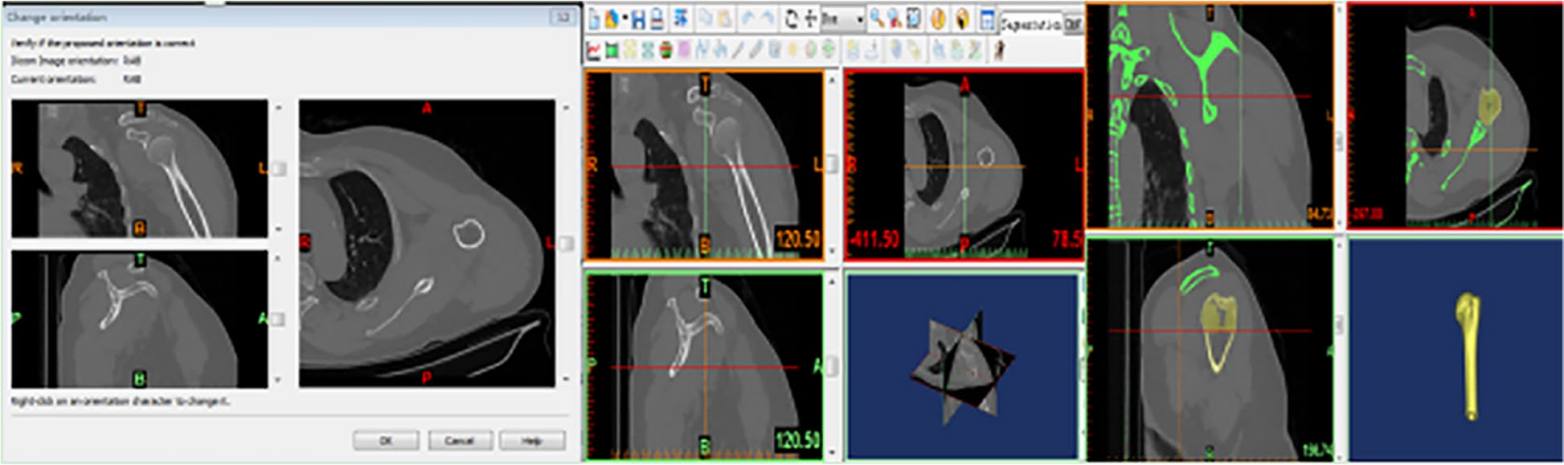
CT image data was imported into Mimics 17.0 software. Construct a 3D model of the humerus

CT scans of the shoulder joint of 23 healthy elderly people (14 males and 9 females, 46 shoulders) were selected from the same CT workstation in the Department of Medical Imaging of Cangzhou Hospital of Integrative Medicine. The orthopedic surgeons examined each case to exclude fractures, deformities, bone tumors, and other lesions. The layer thickness of the spiral CT scan was 1.0 mm, and the image data were imported into Mimics 17.0 software in DICOM format. In the software window, the top, bottom, anterior, posterior, left and right directions were defined. The horizontal, coronal and sagittal images of the acromion and humerus are displayed in three windows. The positioning lines can be moved to observe the different levels (Fig. 2). Select the mask in the toolbar, set the threshold range we need, and click on the region thresholding tool. In any window, select the peak of the shoulder to be reconstructed. Using the “caculate 3D form masks” tool, a 3D model of the acromion will be constructed by performing calculations in the background. Similarly, the humerus is constructed (Fig. 2).

A three-dimensional model was created in Mimics 17.0 software, and the anatomical structures of the proximal humerus were measured. Based on the anatomical data, the acromion and proximal humerus are viewed from different angles and anatomical landmarks are identified: anterior inferior border of the acromion, greater tuberosity, anatomical neck, and medial cortical region of the humeral talus. The parameters measured in this study included: the distance between the inferior border of the acromion and the superior border of the axillary nerve, the distance from the apex of the humeral head to the superior border of the axillary nerve, the vertical distance between the inferior border of the acromion and the superior border of the anterior rotator humeral artery, the vertical distance between the apex of the humeral head and the superior border of the anterior rotator humeral artery, the medial inclination angle (MIA), the superior surface distance (SSD), inferior surface distance(ISD). Medial inclination angle (MIA):The angle between the median axis of the humeral head and the median axis of the humeral shaft in the coronal plane. Superior surface distance (SSD):The projection of the humeral tuberosity in sagittal position as a single line, with the distance between the anterior and posterior cortical boundaries measured at the level of the lowest part of the anatomical neck. Inferior surface distance(ISD):The humeral tuberosity projection in the sagittal position is a single line, and the distance between the anterior and posterior cortical boundaries at the level of 2 cm below the lowest part of the anatomical neck [14].

**Fig. 3:**
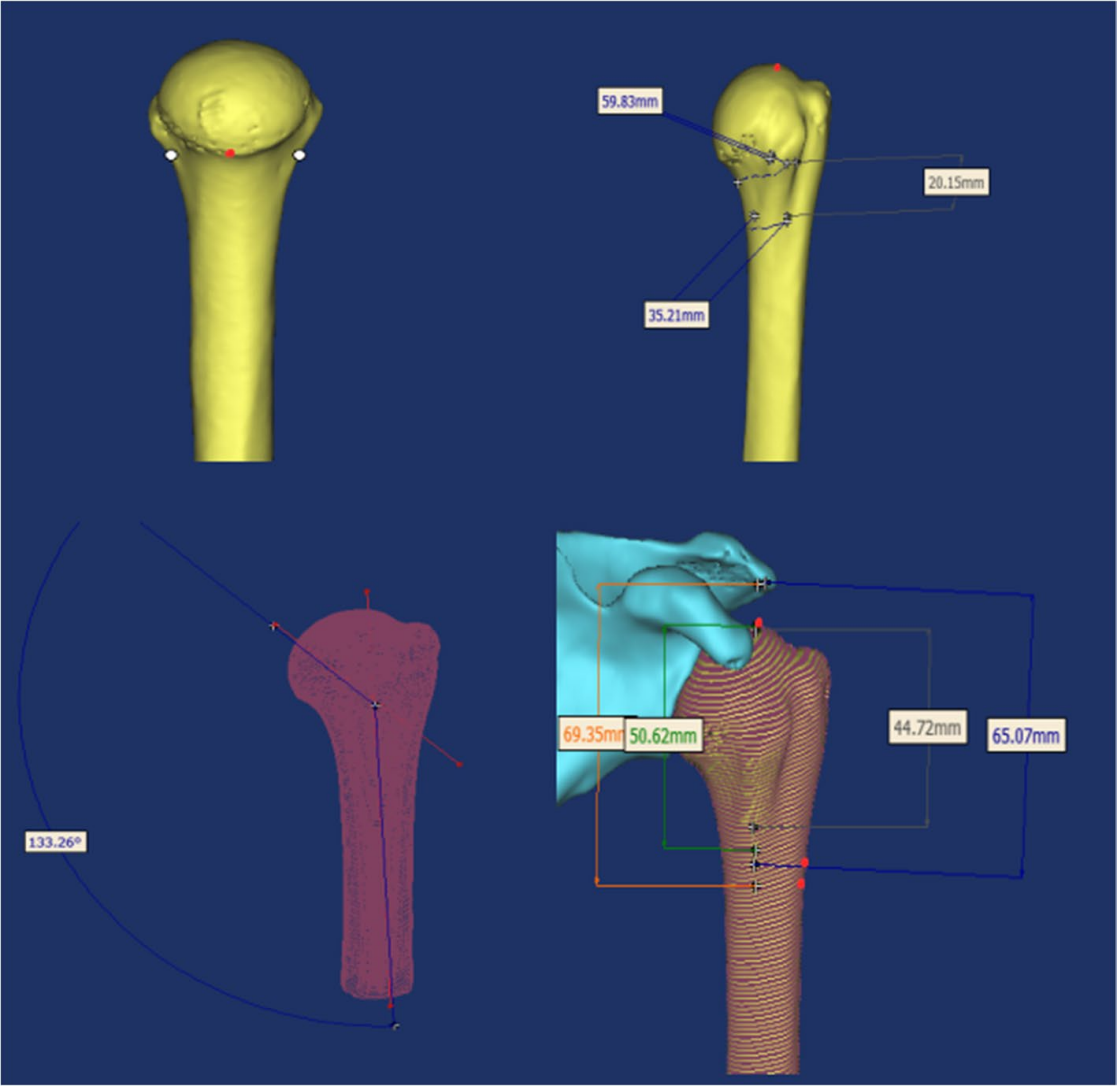
Vertex tag humerus head model, the measurement on the humerus is apart from the surface distance, the distance from the surface of the humeral capitellum and humerus inner Angle, on the basis of anatomical data from measuring mark edge of acromion of depth from the top spin before the brachial artery, humerus head point distance from top spin before the brachial artery, edge of acromion axillary nerves on edge distance, and humerus head point distance from the superior border of axillary nerve

The surgical rediction and fixation of proximal humeral fractures is mainly based on MIA. The anatomical basis for finding the safety zone in the proximal humeral nail placement area was based on seven data: SSD, ISD, vertical distance between the inferior border of the acromion and the superior border of the axillary nerve, vertical distance from the apex of the humeral head to the superior border of the axillary nerve, vertical distance between the inferior border of the acromion and the superior border of the anterior rotator humeral artery, and vertical distance between the apex of the humeral head and the superior border of the anterior rotator humeral artery (Fig. 3).

### Pining point selection and marking

**Fig. 4:**
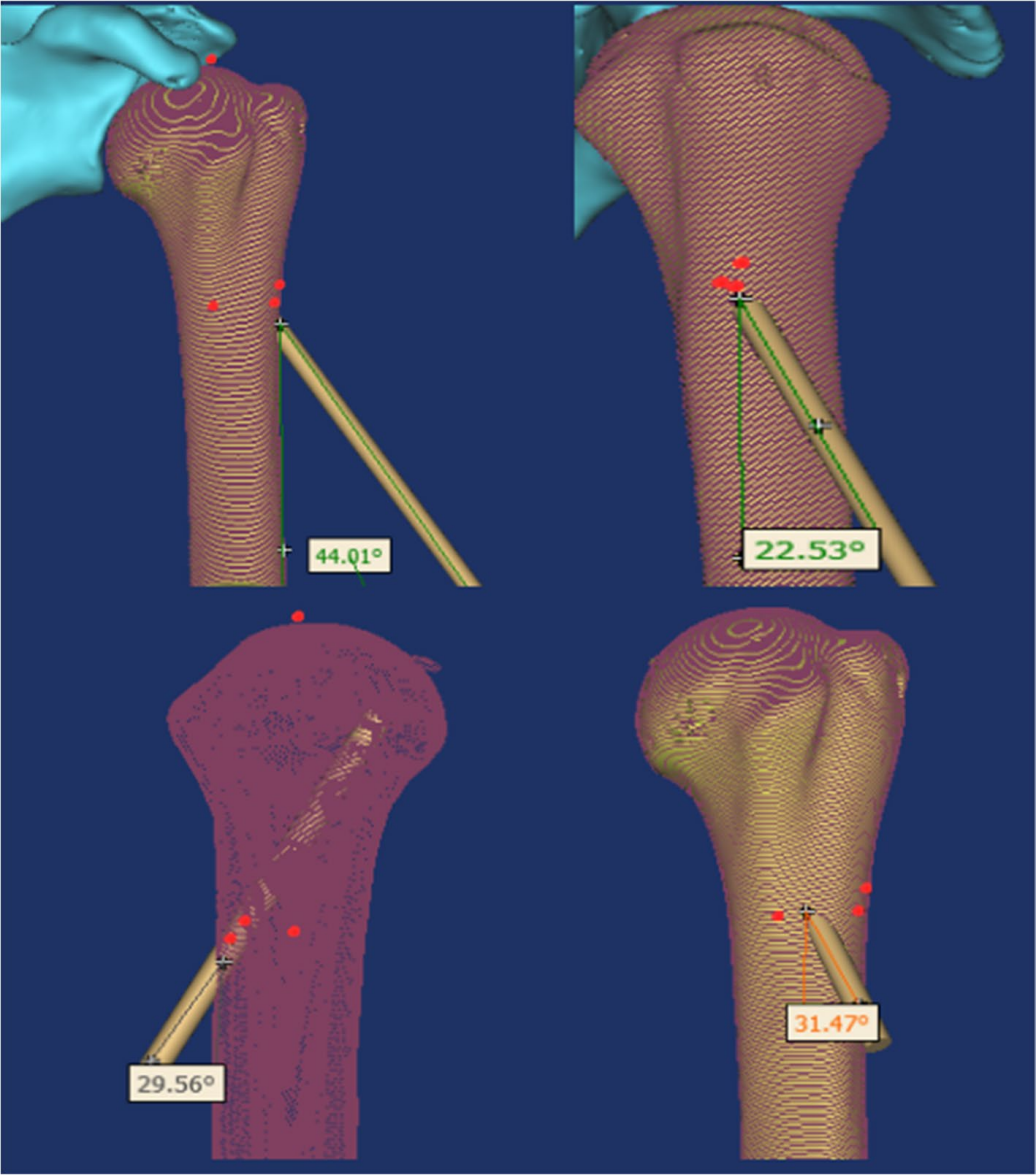
The pining track of the greater tuberosity of humerus and the pining Angle of the coronal plane and sagittal plane of the shaft of humerus

This study focuses on Neer II and III proximal humerus fractures in the elderly. The distance between the inferior border of the acromion and the apex of the humeral head from the proximal axillary nerve of the humerus and the superior border of the spinohumeral artery was measured anatomically, and then the pin placement was simulated by measuring this distance on a three-dimensional model. The angle between the needle and the humeral stem in the coronal and sagittal planes was measured. In general, internal rotation of the humerus during solid patient manipulation will tighten the axillary nerve and the posterior spinohumeral artery and will bring the axillary nerve and the posterior spinohumeral artery closer to the needle position. When the humerus is externally rotated, the axillary nerve and posterior spinohumeral artery will relax and tend to move away from the needle entry position [10] (Fig. 4).

**Fig. 5:**
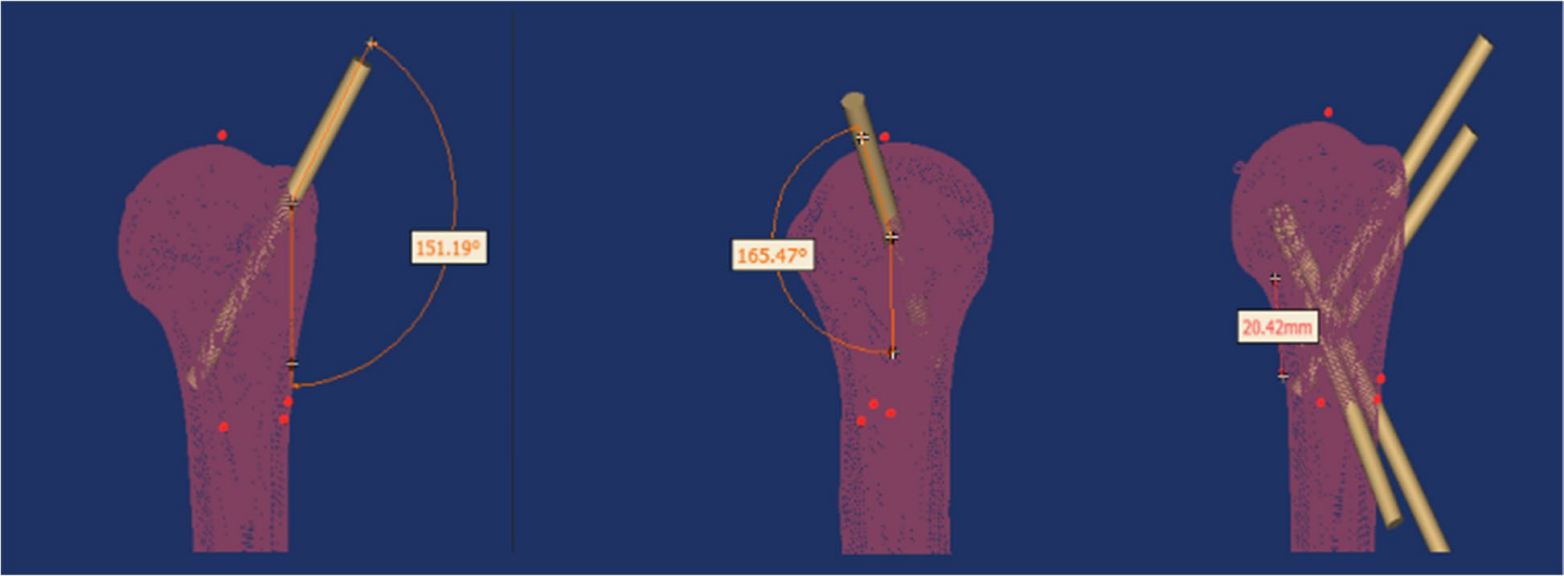
The pining track of the greater tuberosity of humerus and the pining Angle of the coronal plane and sagittal plane of the shaft of humerus

The distance between the inferior border of the acromion and the superior border of the anterior rotator humeral artery, the distance between the apex of the humeral head and the anterior rotator humeral artery, the distance between the inferior border of the acromion and the superior border of the axillary nerve and the distance between the apex of the humeral head and the superior border of the axillary nerve were measured by dissection, and the corresponding positions were marked on the constructed three-dimensional model of the humerus. Using the internal humeral inclination angle as the standard for repositioning, a relatively safe range was found and the pin was threaded along the medial axis of the humeral head, close to the medial cortex of the humeral head to provide neck support. A position is marked on the anterior side of the same plane as the lateral entry point and the pin is cross-placed to fix the humeral head end. The point of entry and the angle of entry are measured. The greater tuberosity of the humerus is fixed with two pins in parallel (Fig. 5).

### Statistical analysis

All statistical data were statistically analyzed using the statistical software SPSS 20.0 (Statistical Package for Social Sciences, SPSS Inc., Chicago, IL, USA) to calculate the results of each measure, and the values are expressed as mean ± standard deviation. Count data were analyzed by t-test, and *P* < 0.05 was set as a statistically significant difference.

## Result

There was no statistically significant difference between the two groups compared to the general data of gender, age, and BMI (p>0.05) (Table 1).

**Table 1:** Cadaversand CT Scan patients characteristics

Characteristics	Gender		Age(year)	BMI (Body Mass Index)
	Male	Female		
Cadavers(*n* = 11)	5	6	67.72 ± 12.36	24.11 ± 2.23
CT Scan patients (*n* = 23)	10	13	65.67 ± 13.64	23.86 ± 3.01
X^2^/t	2.349		4.598	6.783
*p*	0.785		0.415	0.217

### Anatomical results

Twenty-two shoulder specimens of the spinohumeral anterior artery and axillary nerve were completely isolated from the surrounding soft tissue. (1) males: the vertical distance from the inferior border of the acromion of the left shoulder to the superior border of the anterior spinohumeral artery was (5.52 ± 0.73) cm, range (4.6-6.5) cm, and the vertical distance from the inferior border of the acromion of the right shoulder to the superior border of the anterior spinohumeral artery was (5.33 ± 0.66) cm, range (4.5-6.1) cm, with no statistical difference (*p* = 0.636); the left humeral head apex -rotator anterior humeral artery distance was (3.63 ± 0.39) cm, range (3.1 ~ 4.2) cm, right humeral head apex-rotator anterior humeral artery distance was (3.41 ± 0.40) cm, range (2.8 ~ 3.8) cm, no statistical difference (*p* = 0.329); left shoulder subacromial crest-superior axillary nerve margin distance was (5.91 ± 0.62) cm, range (5.2 ~ 6.9) cm, the right shoulder subacromial crest-superior axillary nerve distance was (6.13 ± 0.32) cm, range (5.7 ~ 6.7) cm, no statistical difference (*p* = 0.376); the left humeral head apex-superior axillary nerve distance was (3.92 ± 0.55) cm, range (3.4 ~ 5.0) cm, the right humeral head apex-superior axillary nerve distance was (4.16 ± 0.45) cm, range (3.7 ~ 4.8) cm, no statistical difference (*p* = 0.409) (Table 2). (2) females: the vertical distance between the inferior border of the clavicular end of the left shoulder acromion and the superior border of the anterior rotator humeral artery was (5.82 ± 0.41) cm, range (5.3 ~ 6.3) cm, and the vertical distance between the inferior border of the clavicular end of the right shoulder acromion and the superior border of the anterior rotator humeral artery was (5.63 ± 0.56) cm, range (5.1 ~ 6.2) cm, no statistical difference (*p* = 0.616); the left humeral head apex -rotator anterior humeral artery distance was (3.73 ± 0.59) cm, range (3.0 ~ 4.4) cm, right humeral head apex-rotator anterior humeral artery distance was (3.55 ± 0.73) cm, range (2.8 ~ 4.4) cm, no statistical difference (*p* = 0.722); left shoulder subacromial crest-superior axillary nerve margin distance was (5.73 ± 0.54) cm, range (5.2 ~ 6.3) cm, the right shoulder inferior acromion-superior axillary nerve distance was (5.91 ± 0.23) cm, range (5.7 ~ 6.1) cm, no statistical difference (*p* = 0.512); the left humeral head apex-superior axillary nerve distance was (3.65 ± 0.25) cm, range (3.4 ~ 4.0) cm, the right humeral head apex-superior axillary nerve distance was (3.83 ± 0.33) cm, range (3.4 ~ 4.2) cm, no statistical difference (*p* = 0.432) (Table 3).

**Table 2:** 7 male specimens, the anatomical index distance measurement and statistical analysis results of inferior border of the acromion to the superior border of the anterior rotator humeral artery, apex of the humeral head to the superior border of the anterior rotator humeral artery, inferior border of the acromion to the superior border of the axillary nerve,apex of the humeral head to the superior border of the axillary nerve

Specimen number	Distance from the inferior border of the acromion to the superior border of the anterior rotator humeral artery (cm)		Distance from the apex of the humeral head to the superior border of the anterior rotator humeral artery (cm)		Distance from the inferior border of the acromion to the superior border of the axillary nerve (cm)		Distance from the apex of the humeral head to the superior border of the axillary nerve (cm)	
	left	right	left	right	left	right	left	right
1	5.5	4.8	3.5	2.8	5.7	5.9	3.7	3.9
2	5.4	5.1	3.6	3.3	5.2	5.7	3.4	3.9
3	5.3	5.7	3.4	3.8	6.9	6.2	5.0	4.3
4	6.2	5.9	4.1	3.8	6.1	5.8	4.0	3.7
5	6.5	6.1	4.2	3.8	5.7	6.1	3.4	3.8
6	4.6	4.8	3.1	3.3	5.4	6.3	3.9	4.8
7	5.5	4.9	3.5	3.1	6.1	6.7	4.1	4.7
Mean	5.52	5.33	3.63	3.41	5.91	6.13	3.92	4.16
Standard deviation	0.73	0.66	0.39	0.40	0.62	0.32	0.55	0.45
t	0.486		1.017		0.920		0.856	
p	0.636		0.329		0.376		0.409

**Table 3:** 4 female specimens, statistical analysis results were obtained by measuring the anatomical index distance of anterior circumflex brachial artery of proximal acromion of left and right shoulder humeral, superior circumflex anterior circumflex brachial artery of proximal margin of humeral humeral, superior circumflex anterior circumflex brachial artery of humeral head, superior margin of axillary nerve of inferior margin of humeral shoulder, superior margin of axillary nerve of humeral head

Specimen number	Distance from the inferior border of the acromion to the superior border of the anterior rotator humeral artery (cm)		Distance from the apex of the humeral head to the superior border of the anterior rotator humeral artery (cm)		Distance from the inferior border of the acromion to the superior border of the axillary nerve (cm)		Distance from the apex of the humeral head to the superior border of the axillary nerve (cm)	
	left	right	left	right	left	right	left	right
1	5.7	6.2	3.9	4.4	5.2	5.7	3.4	3.9
2	6.3	5.8	4.4	3.9	5.5	6.1	3.6	4.2
3	5.7	5.2	3.6	3.1	5.7	5.9	3.6	3.8
4	5.3	5.1	3.0	2.8	6.3	5.7	4.0	3.4
Mean	5.82	5.63	3.73	3.55	5.73	5.91	3.65	3.83
Standard deviation	0.41	0.56	0.59	0.73	0.54	0.23	0.25	0.33
t	0.528		0.373		0.697		0.843	
p	0.616		0.722		0.512		0.432	

There were no statistical differences between the left and right sides for four parameters: the distance from the anterior inferior border of the acromion to the superior border of the axillary nerve, the distance from the apex of the greater tuberosity to the superior border of the axillary nerve, the vertical distance from the inferior border of the acromion clavicular end to the superior border of the anterior rotator humeral artery, and the vertical distance from the apex of the greater tuberosity to the superior border of the anterior rotator humeral artery in the same individual. There were no statistical differences between male and female specimens for the four parameters: distance from the anterior inferior border of the acromion to the superior border of the axillary nerve, distance from the apex of the greater tuberosity of the humerus to the superior border of the axillary nerve, vertical distance from the inferior border of the clavicular end of the acromion to the superior border of the anterior rotator humeral artery, and vertical distance from the apex of the greater tuberosity of the humerus to the superior border of the anterior rotator humeral artery (Table 4).

**Table 4:** Statistical analysis distance results of 11 male and female cadaver specimens of anterior circumflex brachial artery of proximal acromion of left and right shoulder humeral, superior circumflex anterior circumflex brachial artery of proximal margin of humeral humeral, superior circumflex anterior circumflex brachial artery of humeral head, superior margin of axillary nerve of inferior margin of humeral shoulder, superior margin of axillary nerve of humeral head

Gender(n)	Distance from the inferior border of the acromion to the superior border of the anterior rotator humeral artery ($\overline{x}$ ±s) (cm)	Distance from the apex of the humeral head to the superior border of the anterior rotator humeral artery ($\overline{x}$ ±s) (cm)	Distance from the inferior border of the acromion to the superior border of the axillary nerve ($\overline{x}$ ±s) (cm)	Distance from the apex of the humeral head to the superior border of the axillary nerve ($\overline{x}$ ±s) (cm)
Male(7)	5.37 ± 0.64	3.52 ± 0.39	5.99 ± 0.46	4.04 ± 0.49
Female(4)	5.6 ± 0.44	3.64 ± 0.62	5.76 ± 0.34	3.74 ± 0.29
t	1.134	0.539	1.188	1.590
p	0.270	0.596	0.249	0.127

### 3D modeling results

46 three-dimensional models of the humerus: (1) male group: left humeral head inversion angle was (134.77 ± 2.67°), range (130.49° ~ 139.78°), right humeral head inversion angle was (134.51 ± 2.35°), range (130.69° ~ 135.84°), no statistical difference (*p* = 0.778); left humeral distance from the upper surface distance was (54.36 ± 2.18°), range (51.87° ~ 59.83°), right humerus distance from the superior surface was (54.45 ± 1.76°), range (51.25° ~ 58.72°), no statistical difference (*p* = 0.905); left humerus distance from the inferior surface was (32.96 ± 1.30°), range (30.78° ~ 35.72°) and the right humeral distance from the inferior surface was (32.89 ± 1.00°) with a range (31.59° to 34.78°), with no statistical difference (*p* = 0.859). (Table 5) (2) Female group: left humeral head internal inclination angle was (132.9 ± 1.08°), range (131.72° to 135.26°), right humeral head internal inclination angle was (132.67 ± 1.26°), range (130.85° to 134.92°), no statistical difference (*p* = 0.565); left humeral distance from the superior surface was (51.81 ± 1.63°), range (49.47°to 54.91°), right humeral distance from the superior surface (51.31 ± 1.06°), range (49.87°to 52.18°), no statistical difference (*p* = 0.444); left humeral distance from the inferior surface (31.75 ± 0.63°), range (30.92° to 32.89 °) and the right humeral distance from the inferior surface was (31.56 ± 0.66°), range (30.18° ~ 32.24°), with no statistical difference (*p* = 0541). There was no statistical difference between the three data of the left and right side humeral head inversion angle, humeral distance from the upper surface of the humeral spine, and distance from the lower surface of the humeral spine in the same body 3D model (Table 6).

**Table 5:** Statistical analysis results of left and right medial inclination angle (MIA) of the humeral head; the superior surface distance (SSD), the inferior surface distance((ISD) in 14 males

Humerus model number	medial inclination angle (MIA) (°)		superior surface distance (SSD) (mm)		inferior surface distanc (ISD) (mm)	
	Left MIA	Right MIA	Left SSD	Right SSD	Left ISD	Right ISD
1	133.26	134.16	55.84	54.93	35.21	34.78
2	132.79	132.34	59.83	58.72	31.84	32.58
3	130.49	131.95	53.12	52.75	32.63	33.04
4	134.61	133.87	55.70	55.31	32.67	33.31
5	135.73	135.84	52.67	53.41	30.78	31.59
6	137.26	137.56	55.41	54.92	32.65	32.23
7	139.78	137.56	54.19	54.41	32.95	32.53
8	134.13	134.11	52.34	53.47	32.42	32.89
9	137.85	136.32	56.33	56.20	32.73	32.14
10	133.98	132.46	52.78	53.64	32.57	31.97
11	134.13	134.98	51.87	51.25	32.46	32.07
12	130.67	130.69	52.94	53.66	34.81	34.22
13	132.83	131.49	53.83	54.92	35.72	34.78
14	136.14	137.12	56.34	56.04	32.56	33.02
Mean	134.77	134.51	54.36	54.45	32.96	32.89
Standard deviation	2.67	2.35	2.18	1.76	1.30	1.00
t	0.285		0.121		0.180	
*p*	0.778		0.905		0.859

**Table 6:** Statistical analysis results of left and right medial inclination angle (MIA) of the humeral head; the superior surface distance (SSD), the inferior surface distance((ISD) in 9 females

Humerus model number	medial inclination angle (MIA) (°)		superior surface distance (SSD) (mm)		inferior surface distanc (ISD) (mm)	
	Left MIA	Right MIA	Left SSD	Right SSD	Left ISD	Right ISD
1	132.72	133.24	51.75	50.27	31.79	30.18
2	132.15	131.86	52.87	51.61	32.89	31.41
3	132.25	131.94	51.15	50.77	31.23	30.88
4	131.72	130.85	52.65	51.43	31.81	31.65
5	132.64	131.98	49.47	50.85	31.45	32.24
6	133.72	132.85	54.91	53.41	32.45	31.76
7	135.26	134.92	50.21	49.87	31.23	31.78
8	132.78	134.12	52.42	51.37	30.92	31.89
9	133.67	132.23	50.89	52.18	31.96	32.23
Mean	132.99	132.67	51.81	51.31	31.75	31.56
Standard deviation	1.08	1.26	1.63	1.06	0.63	0.66
t	0.588		0.784		0.625	
*p*	0.565		0.444		0.541	

The differences were statistically significant (*p* = 0.001) between the constructed 3D models of the male humeral head inversion angle (134.64 ± 2.48°) and female humeral head inversion angle (132.83 ± 1.15°); the humeral distance from the upper surface of the humeral spine (54.41 ± 1.95°) in males and 51.46 ± 1.36° in females (*p* = 0.000); humeral distance from the lower surface of the humeral spine in men (32.93 ± 1.14°) and women (31.65 ± 0.63°), with a statistically significant difference (p = 0.000) (Table 7).

**Table 7:** Statistical analysis results of 23 cases of left and right of medial inclination angle (MIA) of the humeral head; the superior surface distance (SSD), the inferior surface distance((ISD)

Gender(n)	Medial Inclination Angle (MIA) ($\overline{x}$ ±s) (°)	Superior Surface Distance (SSD) ($\overline{x}$ ±s) (mm)	Inferior Surface Distance (ISD) ($\overline{x}$ ±s) (mm)
Male(14)	134.64 ± 2.48	54.41 ± 1.95	32.93 ± 1.14
Female(9)	132.83 ± 1.15	51.56 ± 1.36	31.65 ± 0.63
t	3.434	5.443	4.342
p	0.001	0.000	0.000

### Placement of pins in a safe position

A factor in placing a percutaneous percutaneous pin in the proximal humerus is to evaluate the peripheral neurovascular structures, so we first studied the anatomical position of the axillary nerve and the spinohumeral artery in the adult population by anatomy. The measurement of relevant data on a 3D model of the humerus based on the anatomical basis is simple and intuitive, allowing multiple simulations of the pin trajectory to find the best solution.

A 3.0 mm diameter pin placement die was selected on the 3D model, and for lateral pin entry, the distance from the axillary nerve at the inferior border of the acromion was selected to be (5.90 ± 0.43) cm, range (5.3-6.9) cm, and the pin was entered along the medial axis of the humeral head, close to the medial cervical cortex, and the pin entry angle was measured to be (42.84 ± 2.45)°, range (37.02° ~ 46.31°) in the coronal plane, and (28.24 ± 2.25)°, range (19.22° ~ 28.51°) in the sagittal plane. The pin-in angle was (28.24 ± 2.25)°, with a range of (19.22° ~ 28.51°). The pin was advanced laterally in front of the same level of the lateral approach point, forming a cross-fixed support with the lateral pin, and the pin angle was measured in the coronal plane (36.14 ± 1.75)°, range (30.32° ~ 39.61°), and in the sagittal plane (28.64 ± 1.37)°, range (22.82° ~ 32.11°). Two pins were taken at the greater humeral tuberosity for fixation, with the proximal pin at an angle (159.26 ± 1.98) to the coronal surface of the humeral stem, range (155.79° ~ 165.08°), and the sagittal angle (161.76 ± 2.15)°, with the pin end between the superior surface of the humeral talus and the inferior surface of the humeral talus. The distal needle of the greater humeral tuberosity was parallel to the proximal approach trajectory, and the needle end was on the inferior surface of the humeral talus (Tables 8 and 9).

**Table 8:** Statistical analysis distance results of 11 cadaver specimens of inferior border of the acromion to the superior border of the anterior rotator humeral artery, apex of the humeral head to the superior border of the anterior rotator humeral artery, inferior border of the acromion to the superior border of the axillary nerve,apex of the humeral head to the superior border of the axillary nerve

Anatomical indicators	$\overline{x}$ ±s (cm)	Min-Max (cm)
Distance from the inferior border of the acromion to the superior border of the anterior rotator humeral artery	5.48 ± 0.58	4.8-6.5
Distance from the apex of the humeral head to the superior border of the anterior rotator humeral artery	3.56 ± 0.48	2.8-4.4
Distance from the inferior border of the acromion to the superior border of the axillary nerve	5.90 ± 0.43	5.3-6.9
Distance from the apex of the humeral head to the superior border of the axillary nerve	3.93 ± 0.45	3.4-5.0

**Table 9:** Statistical analysis of the angle between the lateral nail and the humeral shaft, the angle between the anterolateral nail and the humeral shaft, and the angle between the greater tuberosity of the humerus and the humeral shaft in a three-dimensional model of the humerus

Item	$\overline{x}$ ±s (°)	Range (°)
Angle between the lateral nail and humeral shaft		
Coronal surface	42.84 ± 2.45	37.02 ~ 46.31
Sagittal plane	28.24 ± 2.25	19.22 ~ 28.51
Angle between the anterior nail and humeral shaf		
Coronal surface	36.14 ± 1.75	30.32 ~ 39.61
Sagittal plane	28.64 ± 1.37	22.82 ~ 32.11
Angle between humeral greater nodule and humeral shaft		
Coronal surface	148.26 ± 1.98	144.79 ~ 155.08
Sagittal plane	161.76 ± 2.15	159.29 ~ 167.58

## Discussion

The location of the pin placement and the trajectory of the needle insertion depend mainly on the degree of displacement of the fracture end and the fracture line alignment at the cephalic junction [15, 16].Williams [17] suggested that the needle be inserted above the anterolateral stop of the deltoid muscle, with the needle at 45° to the median axis of the humeral stem in the coronal plane and at 30° to the median axis of the humeral stem in the sagittal plane, with the second needle placed parallel to the proximal or distal end of the first needle. The measured angle of needle entry after our simulated pin placement is similar to this result, but it does not guarantee that this is the best solution, taking into account the actual situation of the patient, such as the direction of fracture line extension and the size of the fracture mass. Rowles [16] investigated the placement of pins around the neurovascularization of the proximal humerus. For surgical neck fractures of the humerus, most orthopaedic surgeons consider the application of two anterolateral percutaneous pins for fixation to be essential, but the need for additional pin fixation is not yet clear. Carlo suggests that elderly patients with proximal humeral fractures should be alerted to brachial artery injury and upper extremity deep vein thrombosis [12]. Naidu [18] attempted to answer this question with a cadaveric biomechanical study and found that fixation of the greater tuberosity with only two anterolateral pins was the weakest biomechanical configuration. However, in the study by Ramin [19]. it was found that no failure of internal fixation occurred with fixation of the greater humeral tuberosity with only two anterolateral pins. Jiang [20] compared the biomechanical strength of fixation of the greater tuberosity of the humerus with 4 parallel pins versus 4 converging pins, and they found that parallel pin fixation was biomechanically stronger, but the clinical relevance of this study is questionable because it is quite difficult to insert 2 anterolateral pins parallel to each other in the clinic, let alone 4. The distal two needles were the most difficult to place relative to the placement of the proximal greater and lesser nodal needles. Ramin [19] suggested that the distal two pins be threaded through the proximal humerus anteriorly and laterally, as they simulated that placing the pins at 90° perpendicular to the humeral stem did not provide sufficient holding power. The insertion of the two distal pins is a challenging operation; the average diameter of the adult humeral stem is quite narrow, making it difficult to control the direction of the coronal and sagittal approaches and therefore to take into account the inherent retroversion of the proximal humerus [21]. What we have adopted is the application of two parallel lateral pins in fixation of the greater tuberosity fracture of the humerus.

This study has its own limitations. The sample size of this study was small, and the model constructed was mainly for Neer II and III proximal humerus fractures in the elderly, and other types of proximal humeral fractures in elderly patients were not analyzed, so the scope of application was narrow. Since the model was constructed by inputting CT data into the software, there were unavoidable errors between the model and the real individual. The pin-piercing protocol is simple and easy to perform on the constructed model, but if it is actually performed physically in the clinic, it may be more demanding for the operator, and it may not be possible to completely avoid damage to the anterior rotator humeral artery and axillary nerve, or damage to important vascular nerves due to anatomical variation among individuals.

## Conclusion

In this study, the four data measured in the anatomical specimen: distance of the inferior acromion-superior edge of the axillary nerve, distance of the apex of the greater tuberosity-superior edge of the axillary nerve, vertical distance of the inferior acromion-superior edge of the anterior rotator humeral artery, and vertical distance of the apex of the greater tuberosity-superior edge of the anterior rotator humeral artery were not statistically different in terms of gender and left and right sides of the same body, and for patients with proximal humeral fractures, the same measurement data could be used for pin entry in both men and women The location of the pin was marked. There were no statistical differences between the left and right sides in the three data of the medial inclination angle (MIA) of the humeral head; the superior surface distance (SSD), theinferior surface distance((ISD) of the 3D model of the proximal humerus in the same body, and the data from the contralateral side could be used to guide the repositioning and fixation of the proximal humeral fracture on that side, and uniform data could not be used between male and female patients.

Based on the experimental results obtained above, we found a relatively safe external fixation solution for Neer II and III proximal humerus fractures in the elderly.

## Data Availability

The patients’ dataset are confidential and are privately held for patients confidentiality safeguard. As such, the datasets generated and/or analysed during the current study are not publicly available but are available from the corresponding author on reasonable request.
